# Evaluation of lactic acid as a novel fixative for histological and neuroanatomical applications

**DOI:** 10.1038/s41598-026-51513-y

**Published:** 2026-05-11

**Authors:** Marzia Tindara Venuto, Zinaida Soldat-Böttcher, Julia Kleine, Philipp Warnke, Markus Kipp, Jonas Keiler

**Affiliations:** 1 Institute of Anatomy, Rostock University Medical Center, Gertrudenstrasse 9, 18057 Rostock, Germany; 2 Institute for Medical Microbiology, Virology and Hygiene, Rostock University Medical Center, Schillingallee 35, 18057 Rostock, Germany

**Keywords:** Histology, Fixation, Mouse brain, Neutral buffered formaldehyde, Paraffin sections, Tissue preservation, Biological techniques, Medical research, Neurology, Neuroscience

## Abstract

Formaldehyde-based fixatives, particularly neutral-buffered formalin (NBF), are widely used in histology owing to their strong protein cross-linking capacity and reliable preservation of cytoarchitecture. However, formaldehyde is toxic and carcinogenic, motivating the search for safer alternatives. Here, we systematically evaluated lactic acid (LA) as a potential formaldehyde-free fixative for murine brain tissues. Brains were fixed either by immersion or by transcardial perfusion followed by immersion using LA at varying concentrations, pH-adjusted LA, or phosphate-buffered saline (PBS), and were compared with NBF. Histomorphological preservation was assessed using qualitative evaluation, semi-quantitative scoring, and quantitative crack-area measurements. Across all conditions, NBF provided the best overall tissue preservation. LA fixation showed clear concentration-dependent effects: low concentrations resulted in poor structural integrity, whereas higher concentrations (LA10–LA20) achieved moderate preservation of cytoarchitecture, reduced cracking, and acceptable cellular morphology. White-matter integrity remained inferior to NBF, consistent with the lipid-rich composition of myelin and the predominantly protein-denaturing, non-cross-linking fixation properties of LA. pH-adjustment experiments indicated that fixation efficacy partially depended on acidity. Notably, LA20 delivered by transcardial perfusion substantially improved tissue preservation compared with immersion alone and outperformed PBS. Although LA cannot fully replace NBF, high-concentration LA might represent an alternative for selected neurohistological applications.

**Supplementary Information:**

The online version contains supplementary material available at 10.1038/s41598-026-51513-y.

## Introduction

Tissue fixation is a critical step in histological workflows, aiming to prevent autolysis and degradation. Adequate preservation of tissue integrity is essential to ensure reliable downstream processing and accurate microscopic assessments. Numerous studies have demonstrated that tissue processing—particularly the choice of fixation protocol—profoundly influences the preservation of histomorphology, i.e., the microscopic architecture and organization of cells and extracellular structures^1^.

For decades^2,3^, 10% formalin—corresponding to approximately 3.7% formaldehyde (FA) in a neutral buffered solution, commonly referred to as neutral buffered formalin (NBF)—has served as the standard fixative in routine histopathology. Its widespread use is largely attributable to its reliable preservation of histomorphology coupled with its low cost^4^. FA stabilises tissue architecture by forming covalent cross-links, predominantly methylene bridges, between amino groups^5,6^. This process prevents enzymatic degradation and maintains cellular and extracellular architecture. As a result, FA-fixed tissues remain structurally intact throughout histological processing steps (such as dehydration, clearing in organic solvents, and paraffin embedding), which might otherwise lead to shrinkage or morphological distortions^7^.

However, FA fixation is also associated with methodological limitations, particularly in immunohistochemistry, where cross-linking can lead to epitope masking and reduced antigen detectability.

One of the most severe drawbacks of using FA is its toxicity. FA is a volatile compound that poses significant health risks upon exposure. Inhalation of FA can cause irritation of the skin, eyes, and respiratory tract, leading to diverse symptoms among coughing and throat discomfort, and may result in bronchial inflammation following prolonged exposure^8^. Beyond, recent research has highlighted that long-term exposure to FA is associated with a significantly increased risk of developing nasopharyngeal cancer^9,10^ and leukaemia^11^. Anatomists, histologists, and pathologists are among those most exposed to FA, particularly in anatomical dissection and teaching settings, where prolonged exposure to FA-fixed body donors is common^12,13^. In addition, exposure levels in pathology laboratories have been reported to be substantial^14–16^ indicating that relevant occupational exposure may occur in routine histological workflows. FA exposure also occurs in experimental animal research and broader research environments, reflecting the shared use of FA-based fixatives across these settings, although exposure levels may vary depending on the specific working environment and sample throughput. Accordingly, strict occupational safety regulations have been implemented in histology laboratories and anatomical dissection halls to minimize the exposure risks^17^. Consequently, there is a growing need to explore alternative fixation solutions that provide reliable tissue preservation while reducing potential health risks across different professional and experimental contexts.

In anatomical laboratories, various strategies have already been implemented to reduce FA exposure while maintaining adequate tissue preservation. These include the use of salt-saturated solutions as well as alcohol- and phenol-based fixatives, which have been applied particularly in dissection and teaching settings^18–21^. While these approaches can reduce FA-related exposure, they often involve trade-offs in terms of tissue preservation, handling properties, or long-term stability, and their applicability to routine histological workflows remains limited^22–24^.

In addition to these approaches, a notable number of alternative fixatives have been studied to replace FA in histological and molecular diagnostics^25–27^. Alcohol-based fixatives are known for their ability to precipitate proteins without masking antigenic sites, which typically results in satisfactory histological preservation^27^. However, these fixatives, especially ethanol, often cause significant tissue shrinkage and increased brittleness, which can compromise the quality of the tissue for specific analyses^28^.

Food-grade preservatives represent an attractive alternative for tissue fixation in research, anatomy, and pathology, as they combine antimicrobial properties with low toxicity. Their use could offer a safer and more sustainable approach compared to conventional fixatives. Indeed, natural ingredients such as honey, jaggery, and sugar have long been studied for their preservative properties^29–32^. In addition to these, chemical food preservatives are widely employed when physical methods such as heating, dehydration, or freezing are insufficient to prevent spoilage or degradation. Within the European Union, 42 food preservatives have been officially approved for use. Among them is lactic acid (LA), (designated as E270), which is applied not only in food preservation and processing but also in cosmetics and leather tanning^33^. In addition, LA is incorporated into antibacterial soaps, detergents, and dishwashing products; its antimicrobial activity has been thoroughly investigated^34^. As a naturally occurring organic acid, LA is considered non-toxic, well-studied for efficacy, and is generally recognized as safe for diverse applications^34,35^.

Beyond its preservative function, LA has been shown to exert multiple additional beneficial effects, including the inhibition of autolytic processes, which helps to maintain cellular integrity, as well as antimicrobial properties that contribute to the suppression of bacterial growth^34,35^. Given these characteristics, LA presents itself as a promising candidate for novel applications in tissue preservation, potentially offering an alternative to conventional fixatives such as FA.

In this study, we aimed to evaluate the potential of LA solutions as novel tissue fixatives by assessing their ability to preserve morphological details on the histological level. Particular emphasis was placed on the preservation of cytoarchitecture and suitability for routine haematoxylin-eosin (H&E) staining, as a benchmark for subsequent histological and molecular analyses. We hypothesized that LA solutions could provide fixation effects comparable to those of FA, thereby representing a safer and potentially effective alternative to conventional FA-based fixatives.

Given the distinct requirements of different fixation approaches, we aimed to assess the applicability of LA-based formulations in both immersion-based settings, as commonly used for human brain tissue samples in clinical and research contexts, and perfusion-based approaches, which represent the methodological standard in animal research.

## Results

### Histomorphological comparison of brain tissue preservation after immersion fixation in NBF and LA solutions: descriptive evaluation

**Fig. 1:**
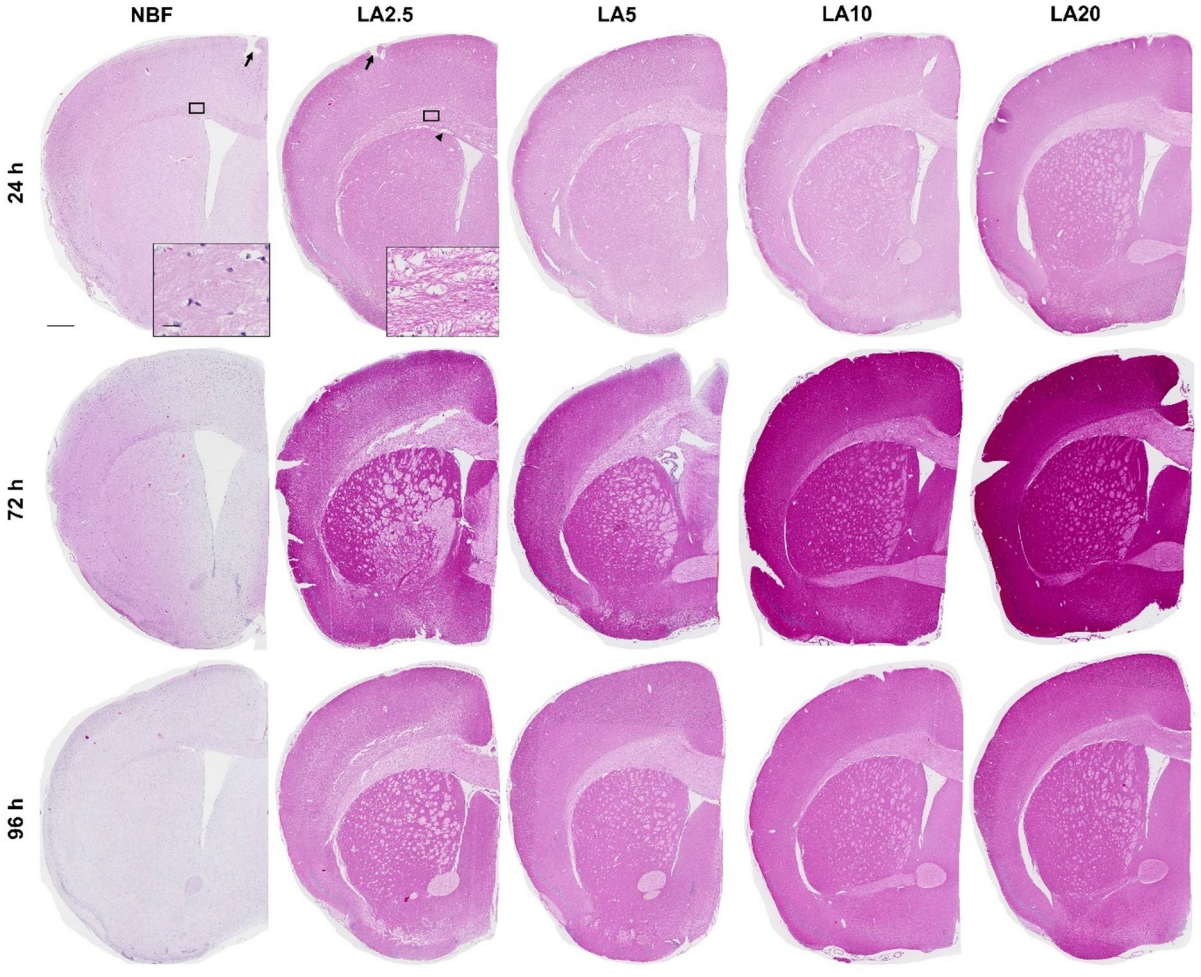
Overview of H&E-stained frontal hemisphere sections (R195–215) illustrating histological tissue preservation in murine brains after immersion fixation with the formalin-based control fixative (NBF) or test fixatives containing lactic acid at different concentrations (LA2.5, LA5, LA10, and LA20). Samples were immersion-fixed for 24, 72, or 96 h (Experiment 1; see Fig. 6 for experimental design). Arrows indicate cracks, which were predominantly observed along the cortical surface. The arrowhead highlights gaps between the corpus callosum white matter and adjacent cortical or subcortical grey matter. Scale bars: overview images, 500 μm; magnified insets, 20 μm.

In the first step, entire brains were immersion-fixed in either NBF or varying concentrations of LA for up to 96 h. The brains were then paraffin-embedded, sectioned, and processed for H&E staining to assess histomorphological quality. Visual inspection under low power magnification revealed that H&E-stained sections from murine brains processed via immersion fixation in NBF showed good histomorphological tissue preservation across all tested immersion durations (24, 72, and 96 h). Overall tissue integrity was well maintained, with only a few to minimal visible cracks. If present, these were predominantly located on the cortical surface (see arrows in Fig. 1). H&E-stained sections from brains fixed for 24 h in NBF exhibited an uniform, moderate eosinophilia, which was slightly reduced after 72 and 96 h of immersion fixation (see Fig. 1). In contrast, and irrespective of LA concentration or immersion duration, H&E-stained sections from brains fixed in LA solutions showed suboptimal histomorphological preservation. Cracks, particularly along the cortical surface, occurred more frequently, and individual axonal fibres running in the white matter appeared separated from each other (compare both inserts in Fig. 1). This was especially obvious within the commissural white matter fibre tract corpus callosum. In some cases, there was an apparent gap between the white matter corpus callosum and the surrounding cortical or subcortical grey matter (see arrowhead in Fig. 1). Furthermore, brain sections fixed for 24 h in LA exhibited moderate eosinophilia, which markedly increased after 72 and 96 h of immersion fixation, resulting in a pronounced demarcation between white and grey matter areas. Nevertheless, H&E-stained sections from brains immersion-fixed in higher LA concentrations displayed a somewhat better histomorphological preservation compared to H&E-stained sections from brains immersion-fixed in lower LA concentrations.

**Fig. 2:**
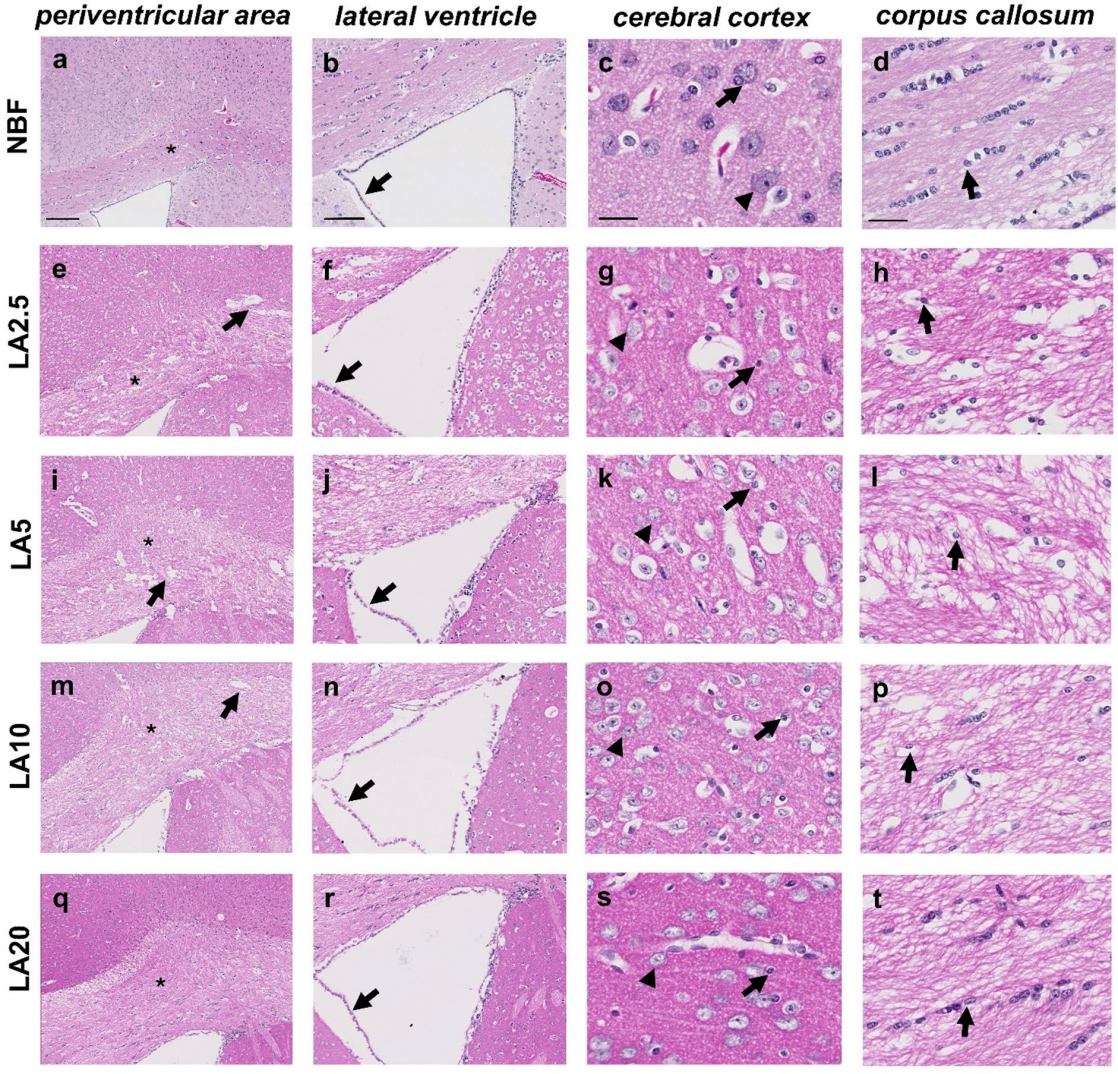
Histomorphological preservation of murine brains after immersion fixation with the formalin-based control fixative (NBF) or test fixatives containing lactic acid at different concentrations (LA2.5, LA5, LA10, and LA20) for 24 h, illustrated by H&E-stained frontal sections (Experiment 1; see Fig. 6 for experimental design). The four columns display: (1) the periventricular area, (2) the lateral ventricle with adjacent corpus callosum and cortical/subcortical grey matter, (3) a high-power view of the cerebral cortex, and (4) a high-power view of the corpus callosum. Stars in a, e, i, m, and q indicate loosening of white matter fibres in the corpus callosum; arrows in b, f, j, n, and r indicate detachment of ventricular ependymal cells; arrowheads in c, g, k, o, and s indicate neuronal cell bodies in the cerebral cortex; arrows in c, g, k, o, and s indicate glial-like cells in the cerebral cortex; arrows in d, h, l, p, and t indicate glial-like cells in the corpus callosum. Scale bars: periventricular area, 200 μm; lateral ventricle, 100 μm; corpus callosum and cerebral cortex, 20 μm. For histomorphology after 72 and 96 h of immersion fixation see Supplemental Files 3 and 4, respectively.

Visual inspection of H&E-stained sections from brains fixed in NBF under high power magnification verified the good tissue preservation. White and grey matter areas were clearly distinguishable, particularly due to the more intense eosinophilia of the white compared to the grey matter (see star in Fig. 2a) and its densely packed and compact organization of myelinated fibre tracts. The ventricular epithelium appeared intact, with no prominent spaces between epithelial cells and the adjacent brain tissue (see arrow in Fig. 2b). Blood vessels were clearly identifiable with minor perivascular spaces (see Fig. 2c). Cells with glial-like morphology were readily identifiable in both the cerebral cortex (see arrow in Fig. 2c) and corpus callosum (see arrow in Fig. 2d), showing well-defined nuclei and only minor pericellular spaces. In addition, cells with astrocyte-like morphology were observed, characterized by oblong, irregularly shaped nuclei. In the cerebral cortex, neuronal cell bodies (see arrowhead in Fig. 2c) exhibited large, nuclei with prominent nucleoli and distinctly defined chromatin patterns. Overall, nuclear morphology was well preserved, showing a distinct basophilic nuclear staining pattern.

Visual inspection of H&E-stained sections from brains fixed in LA solutions under high power magnification confirmed inferior tissue preservation. Several cracks were observed in the corpus callosum (see arrows in Fig. 2e, i, and m), along with larger portions of loosened myelin fibres, particularly at lower concentrations (see Fig. 2h, l, p, and t). The ventricular epithelium showed limited preservation, with spaces between the epithelial cells and adjacent parenchymal tissue (see arrows in Fig. 2f, j, n, and r). Vascular structures appeared poorly defined (see Fig. 2g, k, o, and s). These changes were observed across all LA concentrations; however, they became more pronounced at lower concentrations. Although glial cells and neurons were clearly distinguishable, prominent pericellular spaces were frequently observed, and chromatin organization into euchromatin and heterochromatin appeared less distinct.

### Histomorphological comparison of brain tissue preservation after immersion fixation in NBF and LA solutions: quantitative and semi-quantitative evaluation

So far, the above described observations suggest that LA fixation—particularly at lower concentrations—results in inferior tissue preservation. In the next step, we aimed to (semi-) quantitatively assess tissue preservation. To this end, entire H&E-stained brain sections were digitized, and one hemisphere was defined as the region of interest (ROI). Within this ROI, areas exhibiting insufficient tissue integrity, as indicated by cracks, were manually outlined. Their cumulative area was calculated and expressed relative to the total hemispheric area (referred to as the relative crack area [%]).

**Fig. 3:**
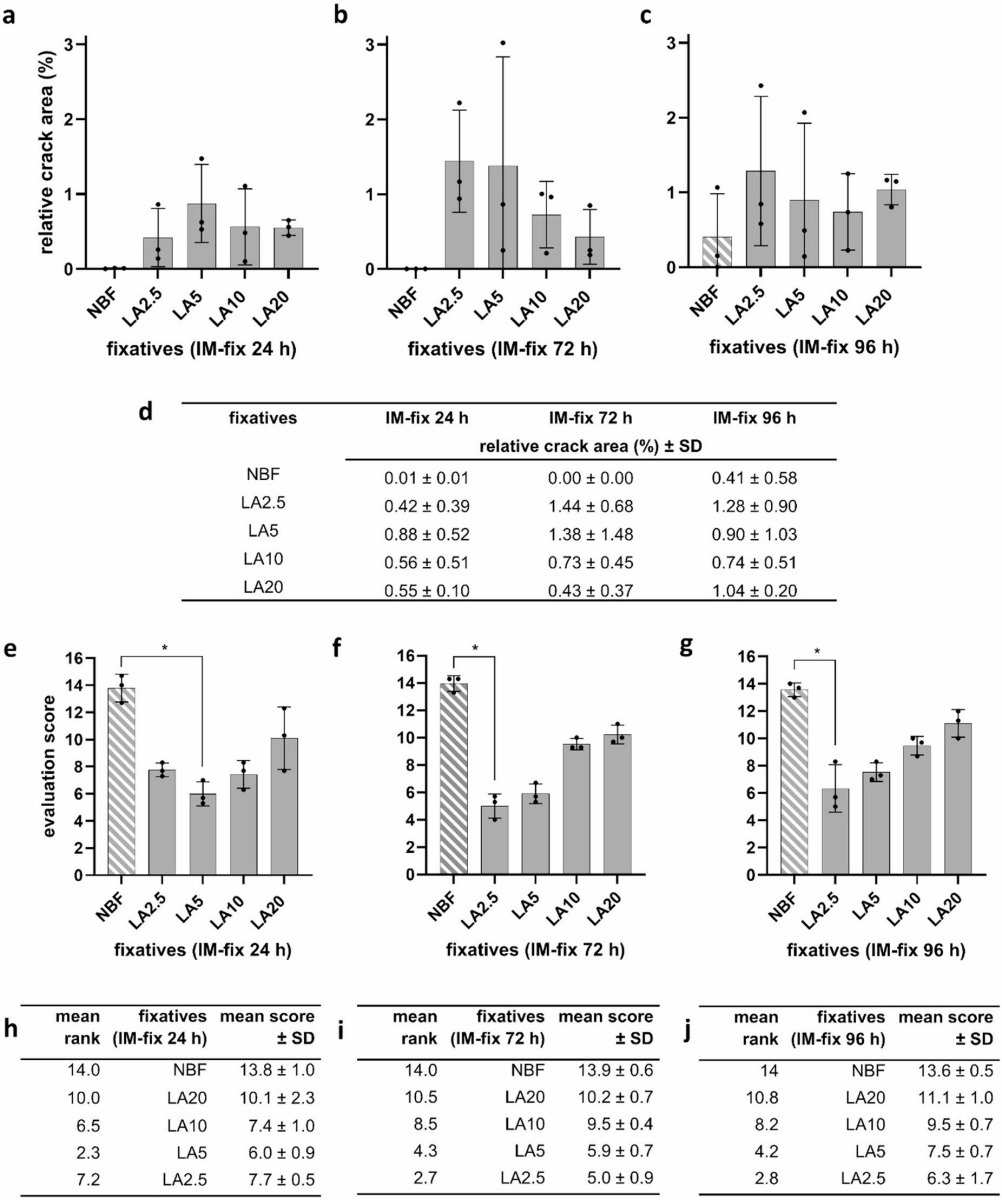
Semi-quantitative assessment of the histomorphological preservation of murine brains after immersion fixation with the formalin-based control fixative (NBF) and test fixatives containing lactic acid at various concentrations (LA2.5, LA5, LA10, and LA20) (Experiment 1; see Fig. 6 for experimental design). (a–c) Bar graphs show the relative crack area ± standard deviation (SD), calculated as the crack area of the hemisphere relative to the total area of the same hemisphere (*n* = 3 hemispheres per fixative) after immersion fixation for (a) 24 h, (b) 72 h, and (c) 96 h. (d) Summary of the relative crack areas ± SD for each group. (e–g) Bar graphs show mean evaluation scores ± SD (*n* = 3 observers, *n* = 3 slides per fixative) after immersion fixation for (e) 24 h, (f) 72 h, and (g) 96 h. Significant differences in evaluation scores were observed between NBF and LA2.5/LA5 (*p* < 0.05). (h–j) Corresponding tables show mean ranks and mean scores ± SD for each group.

Brains immersion-fixed for 24 h displayed crack areas of 0.01 ± 0.01% in the NBF group, 0.42 ± 0.39% in the LA2.5 group, 0.88 ± 0.52% in the LA5 group, 0.56 ± 0.51% in the LA10 group, and 0.55 ± 0.10% in the LA20 group (see Fig. 3a, d; quantification details in Supplemental 1a–c). After 72 h of immersion fixation, no crack areas were detected in NBF-fixed brains, while LA-fixed samples showed 1.44 ± 0.68% (LA2.5), 1.38 ± 1.48% (LA5), 0.73 ± 0.45% (LA10), and 0.43 ± 0.37% (LA20) crack areas (see Fig. 3b, d; Supplemental 1d–f). Following 96 h of immersion fixation, the proportion of cracks within the tissue amounted to 0.41 ± 0.58% in the NBF group, 1.28 ± 0.90% in the LA2.5 group, 0.90 ± 1.03% in the LA5 group, 0.74 ± 0.51% in the LA10 group, and 1.04 ± 0.20% in the LA20 group (see Fig. 3c, d; Supplemental 1 g–i). In summary, across all immersion durations, NBF fixation consistently resulted in the lowest proportion of tissue cracks compared to all tested LA concentrations.

In the next step, we applied a modified scoring system adapted from Kiernan and colleagues^36^ to systematically assess seven histomorphological parameters—cracks, white matter integrity, neuropil precipitates, ventricular epithelial integrity, perineuronal and perivascular spaces, and neuronal nuclear features (nuclear differentiation and chromatin pattern). Each parameter was rated on a three-tier scale reflecting progressive structural deterioration, with a maximum of 16 points indicating optimal tissue preservation.

Brains immersion-fixed for 24 h yielded a score of 13.8 ± 1.0 in the NBF group, 7.7 ± 0.5 in the LA2.5 group, 6.0 ± 0.9 in the LA5 group, 7.4 ± 1.0 in the LA10 group, and 10.1 ± 2.3 in the LA20 group (see Fig. 3e, h; semi-quantification details in Supplemental 2a-e). After 72 h of immersion fixation, brains yielded a score of 13.9 ± 0.6 in the NBF group, while LA-fixed samples yielded a score of 5.0 ± 0.9 (LA2.5), 5.9 ± 0.7 (LA5), 9.5 ± 0.4 (LA10), and 10.2 ± 0.7 (LA20) (see Fig. 3f, i; Supplemental 2a-e). Following 96 h of immersion fixation, brains yielded a score of 13.6 ± 0.5 in the NBF group, while LA-fixed samples yielded a score of 6.3 ± 1.7 (LA2.5), 7.5 ± 0.7 (LA5), 9.5 ± 0.7 (LA10), and 11.1 ± 1.0 (LA20) (see Fig. 3g, j; Supplemental 2a-e).

### LA20 exhibits pH-dependent fixative efficacy superior to PBS but inferior to NBF

Our findings demonstrate that, although LA is less effective than NBF in preserving brain tissue integrity at the histological level, higher LA concentrations produced encouraging results. An important difference between NBF and LA20 is the pH: NBF is neutral (pH 7.4), whereas LA20 is strongly acidic (pH 1.3). To assess the impact of pH on tissue preservation in our experimental setting, both LA20 and PBS were tested in their native and pH-adjusted forms. The pH adjustment was intended to neutralize the acidity of LA20 and to acidify the otherwise neutral PBS, thereby evaluating whether these modifications would enhance or compromise histological preservation. Furthermore, this part of the study aimed to compare the fixative properties of the tested solutions with those of PBS, which served as the vehicle control. Again, we used the evaluation score approach to semi-quantify tissue integrity.

**Fig. 4:**
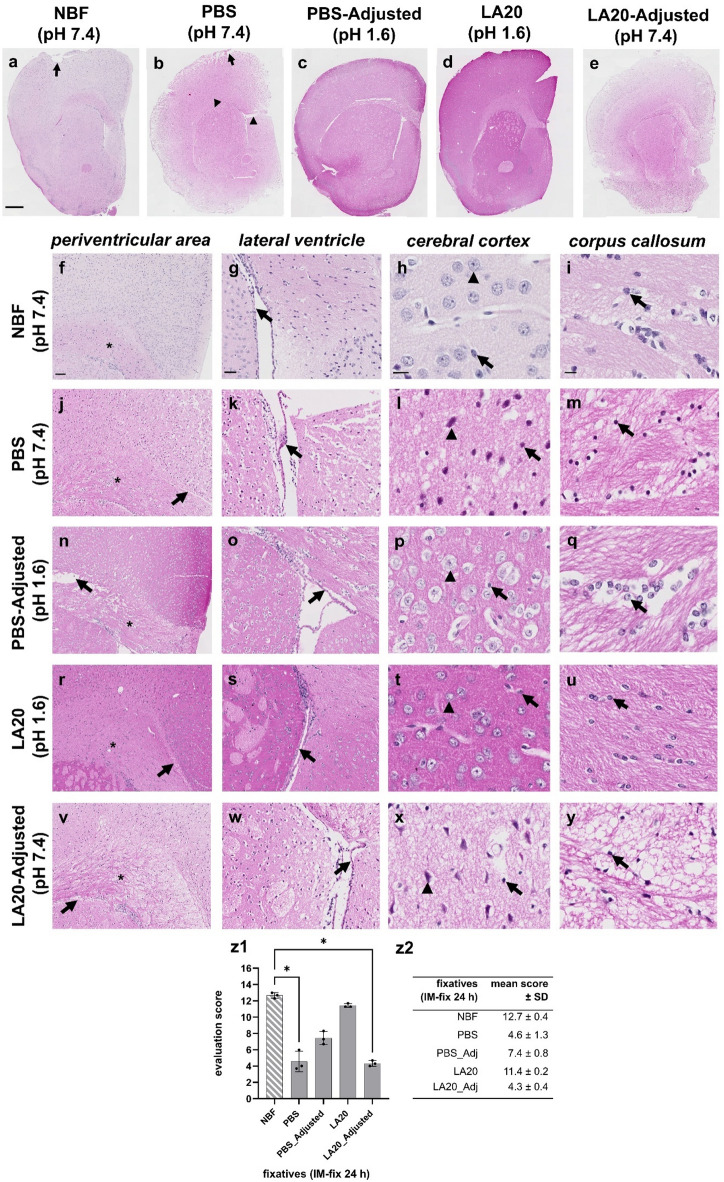
Histomorphological preservation of murine brains after immersion for 24 h with the formalin-based control fixative (NBF) and test fixatives (PBS, PBS-Adjusted, LA20, and LA20-Adjusted) (Experiment 2; see Fig. 6 for experimental design), illustrated by H&E-stained frontal sections (R195) and semi-quantitative evaluation. (a–e) Overview of H&E-stained sections. Black arrows indicate cracks predominantly located on the cortical surface, while black arrowheads mark gaps between the white matter and the surrounding cortical or subcortical grey matter. (f–y) The four columns display: (1) the periventricular area, (2) the lateral ventricle with adjacent corpus callosum and cortical/subcortical grey matter, (3) a high-power view of the cerebral cortex, and (4) a high-power view of the corpus callosum. Stars in f, j, n, r, and v indicate loosening of white-matter fibres in the corpus callosum; arrows in j, n, r and v indicate gaps between the white and grey matter; arrows in g, k, o, s, and w indicate detachment of ventricular ependymal cells; arrowheads in h, l, p, t, and x indicate neuronal cell bodies in the cerebral cortex; arrows in h, l, p, t, and x indicate glial-like cells in the cerebral cortex; arrows in i, m, q, u, and y indicate glial-like cells in the corpus callosum. (z1, z2) Semi-quantitative evaluation scores of the tested formulations. Bar graphs show mean preservation scores (mean ± standard deviation, SD) for each fixative (*n* = 3 observers, *n* = 3 slides per fixative). Significant differences in preservation scores were observed between NBF and PBS, and NBF and LA20_adjusted (*p* < 0.05), respectively. Scale bars: hemisphere overview, 500 μm; periventricular area, 200 μm; lateral ventricle, 100 μm; corpus callosum and cerebral cortex, 20 μm.

Compared to NBF, both native (see Fig. 4b) and pH-adjusted/acidified PBS (see Fig. 4c) resulted in poor histological preservation, characterized by extensive sectioning artifacts, cortical tissue loosening along with white matter cracks. Moreover, there were larger portions of loosened myelin fibres and poorly defined cellular morphologies, which was more evident in the non-adjusted/non-acidified PBS formulation. Consistent with previous findings, LA20 fixation provided moderate preservation (see Fig. 4d), whereas pH adjustment markedly reduced its effectiveness, producing tissue alterations comparable to PBS (see Fig. 4e). NBF again achieved the highest evaluation scores, confirming its superior fixative performance (see Fig. 4z1, z2). More precisely, brains immersion-fixed for 24 h yielded a score of 12.7 ± 0.4 in the NBF group, 4.6 ± 1.3 in the PBS group, 7.4 ± 0.8 in the PBS-Adjusted group, 11.4 ± 0.2 in the LA20 group, and 4.3 ± 0.4 in the LA20-Adjusted group (see Fig. 4z1, z2). These data indicate that the fixative properties of LA20 exceed those of PBS, and that its fixative effect appears to be partially attributable to its low pH.

### Preservation histomorphology effects of LA20 and PBS compared to NBF after transcardial perfusion

While immersion fixation of excised tissue specimens is standard practice in diagnostic pathology, research settings frequently employ transcardial or transvascular perfusion followed by immersion fixation to ensure rapid and uniform tissue preservation. Therefore, we next evaluated the fixation performance of LA under conditions of transcardial perfusion, as illustrated in Fig. 6, Experiment 3.

**Fig. 5:**
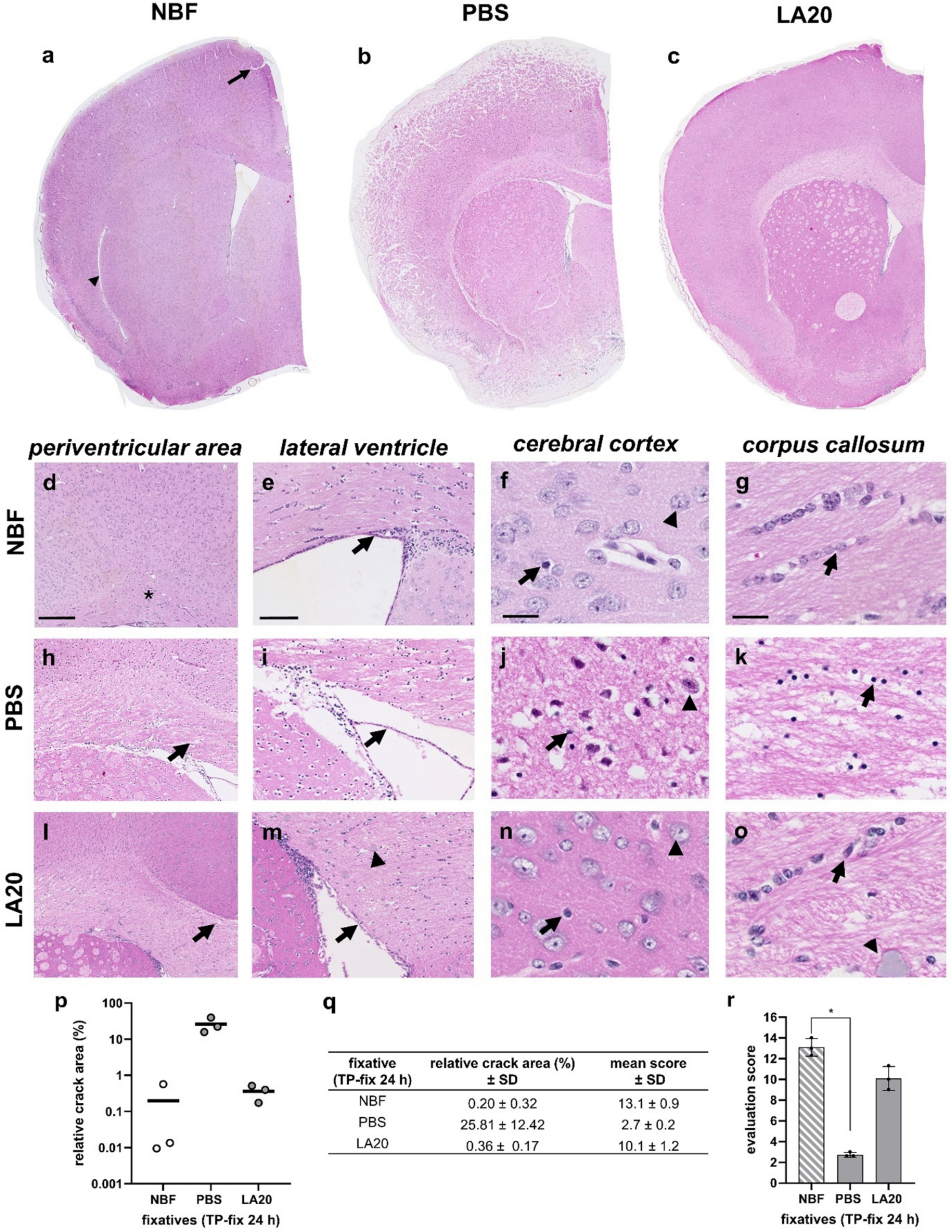
Histomorphological preservation of murine brains after transcardial perfusion, followed by immersion post-fixation for 24 h with the formalin-based control fixative (NBF) or test fixatives (PBS, LA20) (Experiment 3; see Fig. 6 experimental design). (a-c) Overview of H&E-stained sections. Arrow highlights a minor crack located on the cortical surface. (d-o) The four columns display: (1) the periventricular area, (2) the lateral ventricle with adjacent corpus callosum and cortical/subcortical grey matter, (3) a high-power view of the cerebral cortex, and (4) a high-power view of the corpus callosum. Star in d indicates the intact white matter corpus callosum; arrows in h and l indicate loosening of white-matter fibres in the corpus callosum; arrow in e indicates the intact ventricular ependyma; arrows in i and m indicate loosening of ventricular ependymal cells; arrowheads in f, j, and n indicate neuronal cell bodies in the cerebral cortex; arrowheads in f, j and n indicate glial-like cell bodies in the cerebral cortex; arrows in g, k and o indicate glia-like cells in the corpus callosum; arrowheads in m and o indicate precipitates. (p-q) The scatter plot graph represents mean and individual values for the relative crack area (%), calculated as the crack area of the hemisphere relative to the total area of the same hemisphere (*n* = 3 hemispheres per fixatives). (r) The bar graph shows the mean total preservation scores (mean scoring ± standard deviation; *n* = 3 observers, *n* = 3 slides per fixative). Significant scoring differences were observed between NBF and PBS (*p* > 0.05). Scale bars: hemisphere overview, 500 μm; periventricular area, 200 μm; lateral ventricle, 100 μm; corpus callosum and cerebral cortex, 20 μm.

Visual inspection under low power magnification revealed that H&E-stained sections from murine brains fixed by transcardial perfusion using NBF showed good histomorphological tissue preservation (Fig. 5a). Overall tissue integrity was well maintained, with only a few to minimal visible cracks. If present, these were predominantly located on the cortical surfaces (see arrow in Fig. 5a).

The PBS-fixed tissues showed extensive cracks, which were especially evident within the commissural white matter fibre tract corpus callosum and on the cortical surface. H&E-stained sections from murine brains fixed by transcardial perfusion using LA20 exhibited minimal tissue cracks.

Visual inspection under high power magnification of H&E-stained sections from brains fixed in PBS solutions confirmed inferior tissue preservation along with larger portions of loosened myelin fibres (see Fig. 5k). The ventricular epithelium showed prominent spaces between the epithelial cells and adjacent parenchymal tissue (see arrow in Fig. 5i), along with poorly defined cellular morphologies (see Fig. 5j, k).

In comparison, LA20-fixed tissues demonstrated substantially better preservation than PBS, comparable to NBF. Neurons and glial-like cell bodies were clearly distinguishable in the LA20-fixed tissue. Additionally, nuclear architecture was well maintained, with clearly distinguishable euchromatin and heterochromatin, similar to NBF (see arrows and arrowhead in Fig. 5n, o). Interestingly, some precipitates were found in the white matter tract corpus callosum (see arrowheads in Fig. 5m, o).

Quantification of the relative crack area revealed values of 0.20 ± 0.32% in the NBF group, 25.81 ± 12.42% in the PBS group, and 0.36 ± 0.17% in the LA20 group (see Fig. 5p, q). Correspondingly, evaluation scores were low in the PBS group, intermediate in the LA20 group and high in the NBF group (see Fig. 5r, s).

## Discussion

Chemical fixation is a fundamental step to prevent tissue autolysis and degradation while maintaining morphological integrity and cellular detail for microscopic analysis. For more than a century, a 10% formalin solution (3.7% FA), either diluted in water or buffered (i.e. NBF), has been regarded as the fixative of choice in routine histopathology. This preference is largely due to its rapid penetration through cell walls and membranes, its long-term preservation capacity, and its relatively low cost. Formalin induces covalent cross-links between biological macromolecules, thereby stabilizing tissue architecture and preserving both chemical reactivity and antigenicity^37,38^. Consequently, formalin-fixed paraffin-embedded (FFPE) tissues remain the most widely used material for clinical research and molecular diagnostics in pathology laboratories worldwide. However, formalin is not universally optimal, particularly when considering specific downstream biochemical or molecular applications, such as immunohistochemistry or nucleic acid-based analyses, where FA-induced cross-linking may impair antigen accessibility and require adapted protocols for downstream molecular analyses^1,6,27^.

Of note, the present study is limited to the assessment of histomorphological preservation and routine H&E staining, and does not allow conclusions regarding these downstream applications.

In anatomical and experimental settings, several alternative fixation strategies have therefore been developed to reduce FA exposure while maintaining acceptable tissue preservation. These include alcohol-based, phenol-containing, and salt-saturated solutions, which are widely used in anatomical dissection and surgical training. While such approaches can improve handling properties and reduce toxicity, their performance in standardized histological workflows remains variable and has not been consistently evaluated across tissue types and applications.

Phenol-based embalming protocols, for instance, have been reported to provide good macroscopic and cortical preservation, but may exhibit region-specific artefacts, particularly affecting white matter structures, where vacuolization or separation phenomena have been described^22^. Similarly, salt-saturated solution-based fixation approaches can preserve antigenicity but are frequently associated with structural alterations and increased artefact formation in histological preparations^23^.

In addition, strong cross-linking fixatives such as glutaraldehyde are widely used for ultrastructural preservation, but their relatively limited tissue penetration^39^, particularly in larger specimens, may restrict their applicability in routine histological workflows. Moreover, glutaraldehyde is associated with considerable toxicity, including respiratory irritation and sensitization, particularly in occupational settings^40^, which limits its suitability as a general alternative in contexts where reduced FA exposure is desired.

Collectively, these findings highlight that existing FA-reducing or FA-free fixation strategies involve trade-offs between tissue preservation, antigenicity, and structural integrity, and their applicability in standardized histological workflows remains limited. The available literature reflects substantial variability in tissue types, fixation protocols, and evaluation criteria, which further complicates direct comparisons of these approaches.

Within this context, LA-based fixation—particularly at higher concentrations—achieved histomorphological preservation approaching that of NBF in the present study, while still exhibiting limitations, especially in lipid-rich tissues such as white matter.

The present study systematically evaluated the fixation properties of LA-based solutions as potential FA-free alternatives to NBF, the current gold standard in diagnostic neurohistology. Our investigation comprised immersion fixation experiments using a range of LA concentrations, pH-adjusted fixatives, and a direct comparison with transcardial perfusion fixation. The overarching aim was to determine whether LA—particularly at high concentrations—can preserve morphological and cytological integrity in murine brain tissue at levels approaching NBF, while providing a potential alternative in contexts where FA-free fixation is required rather than aiming to universally replace established FA-based protocols.

As expected, NBF consistently produced well-preserved cytoarchitecture with minimal cracking or structural distortions after immersion fixation. NBF-fixed samples consistently exhibited high cytological definition, well-demarcated nuclei, intact ventricular ependyma, and preserved white matter integrity. Of note, the expected temporal dynamics of FA-induced stabilization were evident: during the initial 24–48 h, tissues exhibited minor artifacts consistent with the “half-fixed” state, where diffusion is complete^37,41^ but cross-linking remains immature^42^. By 72–96 h, cross-link formation progressed and cutting quality improved, reflecting the increasing molecular rigidity typical of aldehyde fixation. In comparison, LA fixation produced concentration-dependent preservation quality. Low concentrations (LA2.5 and LA5) were insufficient to stabilize tissue, resulting in extensive crack formation, blurred nuclear morphology, and pronounced structural degradation, especially in white matter–rich regions such as the corpus callosum. Higher concentrations (LA10 and LA20) considerably mitigated these effects and, at short fixation durations, produced morphology that approached NBF. Notably, LA-fixed tissues consistently exhibited enhanced eosinophilia. While this altered staining profile may be disadvantageous for standard H&E interpretation, it also reveals distinct fixation chemistry that could be exploited for specific applications. In this study, we further clarified the underlying mechanisms by examining pH effects. Neutralizing LA20 dramatically reduced preservation capability, rendering tissues morphologically similar to PBS-treated samples. Conversely, acidifying PBS did not improve fixation. These findings suggest that both the intrinsic acidity of LA20 and its propensity to denature and precipitate proteins are contributors to its stabilizing effect, consistent with the established classification of acidic and other non-cross-linking fixatives as acting primarily through protein denaturation and coagulation rather than covalent cross-link formation^43,44^. However, the absence of extensive covalent cross-link formation inherently limits structural rigidity, a defining feature of non-cross-linking fixatives, which may plausibly contribute to persistent artifacts such as edge-associated cracks and partial loss of fine white-matter detail. Perfusion fixation substantially improved preservation across fixatives, highlighting the importance of rapid vascular distribution. LA20-perfused tissue demonstrated markedly better cohesion and clearer cellular profiles than immersion-fixed samples, though NBF still outperformed LA20 in white matter stabilization and nuclear clarity. LA’s lack of protein cross-linking capacity likely contributes to its persistent shortcomings in lipid-rich and structurally delicate regions. Moreover, any stabilizing effect of LA20 may be limited by its acidity, given that the morphological integrity of myelin has been shown to be susceptible to acidic conditions^44^.

In histological preparations of brain tissue, white matter often appears less well preserved than grey matter, likely reflecting the high lipid content of myelin and its susceptibility to fixation- and processing-induced artifacts^45,46^. Our findings in this study are consistent with this observation: whereas high LA concentrations provided acceptable preservation of white matter cytoarchitecture, lower concentrations resulted in multiple fixation artifacts specifically within white matter. This differential preservation likely reflects the distinct chemical and structural properties of both tissue types, combined with the fact that classical fixatives predominantly cross-link proteins, while lipid-rich structures such as myelin are inherently more difficult to stabilize. Grey matter is composed mainly of neuronal cell bodies, dendritic arborizations, numerous synapses, and various glial cell types. These components are rich in proteins and aqueous cytoplasm but comparatively low in lipid-dense myelin. Measurements based on magnetic resonance imaging further demonstrate that grey matter contains approximately 0.83 g H₂O/ml, whereas white matter contains only about 0.71 g H₂O/ml^47^ which likely influences the diffusion efficiency of aqueous fixatives. White matter, in contrast, consists largely of myelinated axons. Myelin comprises multilamellar biomembranes whose dry mass is exceptionally lipid-rich, containing approximately 70–85% lipids and only 15–30% proteins^48^. This unusually high lipid-to-protein ratio underlies both the pale appearance of white matter and the dense, hydrophobic packing of myelin sheaths. Earlier biochemical analyses reported that compact myelin contains approximately 78–81% lipids, whereas the non-myelin components of white matter contain about 49–66%, compared with only 36–40% in grey matter. Major myelin lipids include cholesterol, phospholipids (e.g., plasmalogens), and glycolipids such as galactosylceramide. These biochemical differences have direct implications for fixation efficacy. In grey matter, fixatives primarily interact with water-soluble proteins and nucleic acids, structures readily accessible to aqueous solutions. In white matter, however, fixatives must penetrate thick, hydrophobic, lipid-rich myelin layers, where only minimal protein content is available for covalent cross-linking. This limited accessibility likely contributes to the reduced preservation quality and higher susceptibility to artifacts observed in poorly fixed white matter.

Taken together, these considerations place LA among acidic, non-cross-linking fixatives whose stabilizing effects rely primarily on protein denaturation rather than covalent cross-link formation. While this mechanism appears sufficient for preserving protein-dominated grey matter, it is less effective in lipid-rich tissues such as white matter, where myelin constitutes a major structural component. In this context, the limited stabilization of lipid-rich compartments, combined with partial lipid extraction during downstream processing steps—including washing, dehydration, and embedding—likely contributes to deformation of myelin sheaths and suboptimal preservation of fine axonal architecture. The comparatively lower water content of white matter may further restrict fixative penetration, thereby exacerbating these limitations.

An important aspect not addressed in the present study concerns the preservation of antigens for subsequent immunohistochemical analyses. As outlined above, formalin fixation relies on the diffusion of FA into the tissue, where it reacts with amino acid side chains to form methylene bridge cross-links. While these cross-links effectively preserve morphology, they can also mask epitopes and reduce antigen accessibility, often necessitating antigen retrieval (e.g., heat-induced epitope recovery or proteolytic digestion). To the best of our knowledge, no published data are currently available on antigen preservation following LA-based tissue fixation. Our preliminary observations suggest that immunohistochemistry on LA-fixed, paraffin-embedded tissues yields robust staining for some antigens, whereas others are only weakly detectable. Given that fixation chemistry, additives, and immersion duration can critically influence the visualization of specific cellular and subcellular components^49^, it is conceivable that LA-based fixation may prove particularly suitable for a subset of antigens, while being less effective for others. Further systematic studies will be required to define these antigen-specific differences.

## Conclusion

In summary, LA-based fixation showed concentration-dependent preservation of brain cytoarchitecture, with higher concentrations approaching the performance of NBF under certain conditions. However, NBF remains the gold standard for histological brain tissue fixation, particularly with regard to structural stability and white matter preservation. LA-based fixation may represent a useful FA-free alternative in selected applications, but its limitations, particularly in lipid-rich tissues, must be considered.

## Materials and methods

**Fig. 6:**
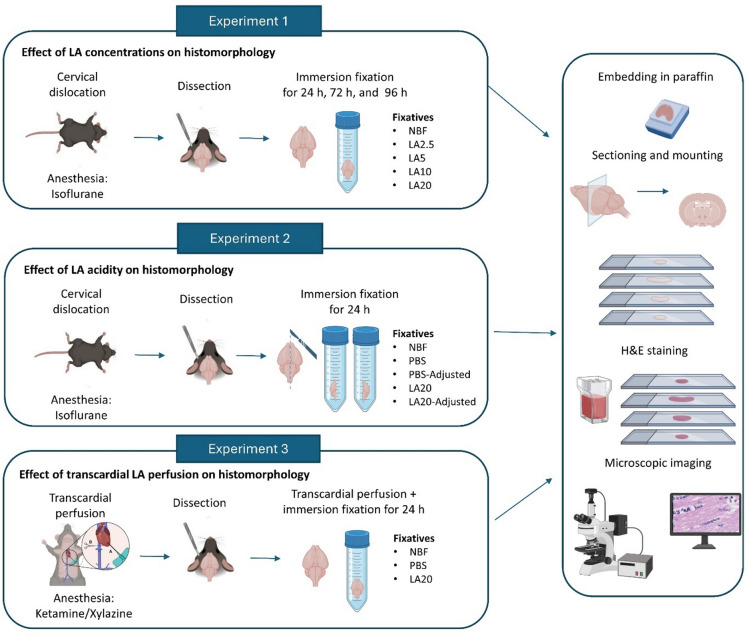
Schematic workflow for the murine brain fixation used in this study. Test solutions based on lactic acid (LA) were evaluated in different experimental setups, including immersion fixation or transcardial perfusion followed by immersion post-fixation. In **Experiment 1**, the effects of varying LA concentrations on histomorphology were assessed at various immersion durations: specimens were sacrificed by cervical dislocation, and whole brains were immersed in control (NBF) or test solutions (LA2.5, LA5, LA10, LA20) for 24 h, 72 h, or 96 h. In **Experiment 2**, the effects of LA acidity were examined: brains from cervically dislocated specimens were sagittally bisected, and hemispheres were separately immersed for 24 h in control (NBF, PBS) or test solutions (PBS_Adjusted, LA20, LA20_Adjusted). In **Experiment 3**, the effects of LA following transcardial perfusion were investigated: animals were perfused with control (NBF, PBS) or test (LA20) solutions, and whole brains were subsequently post-fixed by immersion for 24 h. All samples were paraffin-embedded, sectioned coronally, stained with H&E, and imaged using light microscopy. Figure created with BioRender (BioRender.com) and used with permission.

Murine brains were fixed using various LA formulations followed by analyses of paraffin-embedded sections for histomorphological details. A set of different fixation experiments (Fig. 6) was conducted to investigate,

- if LA concentration influences histological preservation effects under varying immersion durations (Fig. 6, Experiment 1),
- to what degree histological preservation effects relate to the fixative’s pH (Fig. 6, Experiment 2),
- if LA is suitable for histological fixation using transcardial perfusion (Fig. 6, Experiment 3).

### Fixative solutions

For all experiments, NBF with a pH of 7.4 was used as the gold standard, while phosphate buffered saline (PBS) at 0.1 M and with a pH of 7.4 served as non-fixation control. LA was tested in aqueous solutions which contained four concentrations of LA (2.5%, 5%, 10%, 20%) dissolved in PBS (details provided in formulations paragraph) .

Fixation effects related to acidity were studied using a PBS solution acidified to a pH of 1.6, matching the acidity of 20% aqueous LA (for details see formulation paragraph). Furthermore, the fixation effects of LA independent of its acidity were evaluated using an aqueous solution of 20% LA adjusted to a pH of 7.4 (details provided in Supplemental 6, Table 2). All solutions were freshly mixed before use and stored at 4 °C.

#### Formulations

##### NBF

Monosodium phosphate monohydrate (NaH2PO4·H2O), (Merck, Darmstadt, Germany; Cat. No. 1.06346.1000) at 0.46% (w/w) and disodium phosphate dihydrate (Na2HPO4·2H2O) (Merck, Darmstadt, Germany; Cat. No. 1.06580.1000) at 0.80% (w/w) were dissolved in distilled water, followed by the addition of a 37% (w/w) FA solution (Carl Roth, Karlsruhe, Germany; Cat. No. 7398.4) to achieve a final concentration of 3.7% (v/v). The pH was adjusted to 7.4 by the dropwise addition of sodium hydroxide (NaOH, 5 mol/L, Carl Roth, Karlsruhe, Germany; Cat. No. KK71.1) with constant stirring.

##### PBS

Sodium chloride (NaCl) (Carl Roth, Karlsruhe, Germany; Cat. No. 0962.2) at 4.00% (w/w), potassium chloride (KCl) (Carl Roth, Karlsruhe, Germany; Cat. No. 6781.3) at 0.10% (w/w), Na_2_HPO_4_·2H_2_O (Supelco Bellefonte, USA; Cat. No. 1.06580.1000) at 0.84% (w/w), and potassium dihydrogen phosphate (KH_2_PO_4_) (Carl Roth, Karlsruhe, Germany; Cat. No. P018.2) at 0.14% (w/w) were dissolved in distilled water. The pH was adjusted to 7.4 by the dropwise addition of NaOH (5 mol/L, Carl Roth, Karlsruhe, Germany; Cat. No. KK71.1) or hydrochloric acid (HCl, 5 mol/L, Carl Roth, Karlsruhe, Germany; Cat. No. 1E2C.1), respectively, with constant stirring.

The acidified PBS solution (PBS_Adjusted) was prepared by the dropwise addition of HCl with constant stirring to achieve a pH of 1.6.

##### LA

The aqueous LA solutions were prepared using LA (Chemiekontor.de GmbH, Mannheim, Germany; Cat. No.72007106) dissolved in 0.1 M PBS, and diluted to final LA concentrations of 2.5% (v/v) (LA2.5), 5% (v/v) (LA5), 10% (v/v) (LA10), and 20% (v/v) (LA20), respectively (for pH details see Supplemental 6, Table 2).

The pH-adjusted solution of 20% LA (LA20_Adjusted) was prepared by the dropwise addition of NaOH with constant stirring to achieve a pH of 7.4.

### Specimens

Brains from female C57BL/6 mice (Janvier Labs, Le Genest- Saint-Isle, France), aged 9 to 12 weeks, were fixed and analyzed. The animals were housed under standard laboratory conditions following the guidelines of the Federation of European Laboratory Animal Science Associations (FELASA), until the day of euthanasia. For each fixative, three mice were included per group in each experiment. All procedures involving animals were conducted in accordance with FELASA recommendations and approved by the competent local authority (Landesamt für Landwirtschaft, Lebensmittelsicherheit und Fischerei Mecklenburg-Vorpommern; approval no. 7221.3-1-045/23). This study is reported in accordance with the ARRIVE guidelines for reporting animal research.

### Fixation method

The brains were either fixed by immersion (IM-fix) or transcardial perfusion (TP-fix) followed by immersion fixation (Fig. 6).

#### IM-fix

For immersion fixation, animals were anesthetized by inhalation of isoflurane vapor prior to euthanasia. Isoflurane (AbbVie, Ludwigshafen, Germany; Cat. No. B506) was administered via vapor exposure in a closed chamber by placing a cotton swab soaked with liquid isoflurane inside a sealed glass container. Animals were exposed until loss of the righting reflex, after which euthanasia was performed by cervical dislocation. Following euthanasia, the pericranium was removed and the skull was carefully opened. The brains were removed from the skull by severing the medulla oblongata. Once removed, the brains were immersed in 50 ml of the control or test solution at 4 °C. Immersion durations lasted for 24, 72, and 96 h, respectively. For the experiment assessing acidity effects, using the PBS-Adjusted and the LA-Adjusted solutions, the brains were divided sagittally along the longitudinal fissure with a razor blade and the hemispheres were immersed in 50 ml of control or test solution at 4 °C for 24 h. Details of the tested solutions and immersion durations are given in Fig. 6 and Supplemental 6, Table 2.

#### TP-fix

For fixation with transcardial perfusion followed by immersion fixation, animals were deeply anesthetized by intraperitoneal injection of a lethal dose of ketamine/xylazine (100 mg/kg and 10 mg/kg respectively), (Ketamine 10%, HFW- Haupt Pharma Wülfing GmbH, Halle, Germany; 27015.00.00; and Xylazine-Rompun ^®^2%, Leverkusen, Germany, 6293841.00.00) in accordance with FELASA guidelines. After confirming the loss of any heartbeat, the abdomen and thorax were opened. The right cardiac auricle was incised to allow exsanguination, followed by perfusion with 10 mL of chilled PBS through the left cardiac ventricle for approximately 3 min using an automatic rotatory pump (IsmaTech IP-4, Fischer Scientific). Subsequently, perfusion with 40 mL chilled control or test solution was performed for about 10 min. Following perfusion, the cadavers were decapitated, and the brains were removed as described above. The brains were then immersed in 50 mL of the corresponding control or test solution. Details of the tested solutions and immersion durations are given in Fig. 6 and Supplemental 6, Table 2.

After fixation, the samples were rinsed in running tap water for six hours to remove any remaining fixative from the tissues. Following rinsing, the samples were transferred to 50% ethanol (Ethanol 96%, Walter CMP, Kiel, Germany; Cat. No. wai641602) and stored overnight at 4 °C. The samples were then transferred to 70% ethanol (Ethanol 96%, Walter CMP; Cat. No. wai641602) for the following embedding procedures using an automatic embedding machine (Medim, DDM P800). During embedding, the samples were dehydrated through an ascending series of ethanol (80%, 96%, 100%), (Ethanol 99%, Walter CMP; Cat. No. wal6426025) transferred to 100% xylene/xylol (Walter CMP,; Cat. No. wal124020025), and finally embedded in liquid paraffin (Histosec paraffin, Sigma-Aldrich; Cat. No. 1.116.099.025). For dehydration step details, see Supplemental 6, Table 3.

The sample blocks were embedded using a paraffin dispensing station (EG 1150, Leica Biosystems, Wetzlar, Germany), with paraffin (Histosec, Sigma-Aldrich, ; Cat. No. 1.116099.025) maintained at 61 °C. Frontal brain sections with a thickness of 5 μm were obtained using a rotary microtome (RM2277, Leica Biosystems). Sections containing the anterior commissure, corpus callosum, and striatum–corresponding to atlas levels 195–215 according to the anatomical mouse brain atlas by Sidman et al.^50^–were targeted. Multiple pairs of consecutive sections per brain from the targeted region (levels 195–215) were mounted on Superfrost^®^ Plus slides (Epredia, Breda, Netherlands). Frome these, two sections per specimen were selected for histomorphological staining and subsequent analysis, whereas additional sections were reserved for complementary analyses not included in the present study. Mounted slides were air-dried at room temperature for at least 3 h, followed by incubation at 48 °C for 24 h.

### Staining

Prior to staining, sections were deparaffinized in an automated staining system (ST5020, Leica Biosystems) using xylene, followed by rehydration through a graded series of ethanol and immersion in distilled water (see Supplemental 6, Table 4 for details).

H&E staining was performed using Mayer´s Hematoxylin (Sigma-Aldrich, Cat. No. 1.09249.2500) and Eosin-G (Carl Roth, Karlsruhe, Germany; Cat. No. 7089.2) on the same automated system (see Supplemental 6, Table 5, for incubation and differentiation steps). After staining, sections were dehydrated through a graded ethanol series and cleared, followed by automated coverslipping (CV5030, Leica Biosystems) (see Supplemental 6, Table 6, for details). Depex (Serva, Heidelberg, Germany; Cat. No. 18243.02) was used as the mounting medium.

### Evaluation of histomorphological tissue preservation

To compare the fixation efficacy of different fixatives, LA concentrations, immersion durations, and fixation methods, histomorphological and cytological preservation were assessed on the H&E-stained Sect.  (2 sections per brain).

General tissue preservation was first assessed qualitatively using a bright-field microscope (DM6B, Leica Microsystem, Wetzlar, Germany). The stained sections were inspected for overall morphology, tissue integrity, and the presence of fixation-related artifacts, with a particular focus on crack formation.

To complement the qualitative evaluation, crack formation in the sections was quantified using QuPath (v.0.4;^51^. For each fixation condition, unilateral brain hemispheres (1 per section; 2 per brain) were analyzed according to the following workflow:

1. Two sections per brain were digitized using a slide scanner (Grundium Ocus 40, Tampere, Finland), and images were saved as SVS files and opened in QuPath.
2. A region of interest (ROI) corresponding to one hemisphere each was manually outlined using the polygon tool in QuPath; the hemisphere area was then calculated.
3. Within each hemisphere ROI, cracks were identified, manually annotated, and the crack area was calculated. Crack-like distortions overlapping with vacuole formation were not included due to ambiguous attribution.
4. The relative crack area (%) was calculated as the ratio of the mean crack area to the mean hemisphere area:

$$\:relative\:\:crack\:\:area\:\:\left(\%\right)=\frac{crack\:\:area}{hemisphere\:\:area}\:*100$$

In addition, a structured semi-quantitative assessment was performed to complement the descriptive findings. All slides were pseudonymized by assigning random numerical codes prior to evaluation by three independent observers, who were blinded to fixation conditions. The best-preserved section per specimen was selected for scoring, and all observers evaluated the same section.

Tissue quality was assessed at multiple magnifications:

- **4× objective**: general impression, including (subjective) frequency and distribution of cracks (Table 1, Sect.  1).
- **20× objective**: histomorphology of key anatomical regions (Table 1, Sect.  2).
- **40× objective**: cytological features (Table 1, Sect.  3).

Tissue preservation was scored according to an expanded version of the system by Kiernan *(26)*:

- **2 = Good preservation**.
- **1 = Intermediate preservation**.
- **0 = Poor preservation**.

Each observer independently scored all features. Feature-level scores were averaged across observers and then summed to obtain a total preservation score for each section. Group-level scores were calculated as means of the individual section scores. Total scores ranged from 0 (poor preservation) to 16 (excellent preservation).

**Table 1:** Semi-quantitative scoring system for assessing histomorphological features in murine brain sections to evaluate fixation quality.

	score
**(1) General impression**	**0-2**
**Cracks**	
Section without cracks	2
Section with occasional cracks	1
Section with abundant cracks	0
**(2) Histomorphology**	**0–10**
**White matter integrity (corpus callosum)**	
Fibres in Corpus Callosum densely packed	2
Fibres in Corpus Callosum in intermediate condition	1
Fibres in Corpus Callosum largely loosened	0
**Neuropil precipitates (white matter)**	
No precipitates present	2
Occasional precipitates	1
Abundant precipitates	0
**Integrity of the ventricular epithelium (ependymal cells)**	
Epithelium intact, not torn, and no spaces between the ventricular epithelium and adjacent brain tissue	2
Epithelium in intermediate conditions (some spaces between epithelium and adjacent tissues)	1
Epithelium extensively torn or with prominent spaces between epithelium and adjacent brain tissue	0
**Perineuronal spaces (cortex)**	
No perineuronal spaces present	2
Visible but narrow perineuronal spaces (less than width of cytoplasm between nucleus and plasma membrane)	1
Wide perineuronal spaces (equal or greater than diameter of the nucleus of a large neuron)	0
**Perivascular spaces (cortex)**	
Perivascular spaces not visible	2
Visible but narrow spaces (less than half the diameter of the vessel)	1
Large spaces (equal or greater than diameter of the vessel)	0
**(3) Cytology**	**0–4**
**Cell nuclei differentiation (cortex)**	
Nuclei of neurons and glial cells- like can be differentiated	2
Nuclei of neurons and glial cells- like in intermediate condition	1
Cell nuclei appear homogeneous, cell types cannot be differentiated	0
**Neuronal chromatin pattern (cortex)**	
Neuronal chromatin pattern (euchromatin and heterochromatin) clearly visible	2
Neuronal chromatin pattern only partially visible (absent in some cells and/or only partially present in others)	1
Neuronal chromatin pattern not visible, nuclei uniformly stained	0

### Statistical analyses

All data were processed and analyzed using GraphPad Prism software (Boston, Massachusetts, USA; version 8.0.2). Differences between fixation groups were assessed with the Kruskal–Wallis test, followed by Dunn’s multiple comparisons test. Pairwise comparisons between individual groups were performed using the unpaired Mann–Whitney U test. Statistically significance was set at *p* < 0.05.

## Electronic Supplementary Material

Below is the link to the electronic supplementary material.

Supplementary Material 1

Supplementary Material 2

Supplementary Material 3

Supplementary Material 4

Supplementary Material 5

Supplementary Material 6

Supplementary Material 7

Supplementary Material 8

## Data Availability

The data that support the findings of this study are available on request from the corresponding author.
