# Supplementary material for: Evaluation of lactic acid as a novel fixative for histological and neuroanatomical applications
Source: Sci Rep. 2026 May 11;16:15746. doi: 10.1038/s41598-026-51513-y (PMC13190837; doi:10.1038/s41598-026-51513-y)
Supplement: Supplementary file 1 — Supplementary Material 1 [file 41598_2026_51513_MOESM1_ESM.pdf]

a b

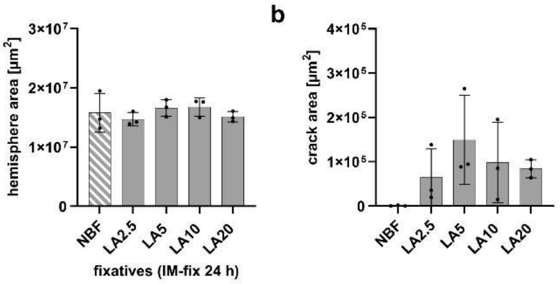

c

fixative (IM-fix 24 h)	hemisphere area (µm²) ± SD	crack area (µm²) ± SD
NBF	$1.58 \times 10^7 \pm 0.33 \times 10^7$	$0.01 \times 10^5 \pm 0.01 \times 10^5$
LA2.5	$1.47 \times 10^7 \pm 0.11 \times 10^7$	$0.64 \times 10^5 \pm 0.64 \times 10^5$
LA5	$1.66 \times 10^7 \pm 0.14 \times 10^7$	$1.49 \times 10^5 \pm 1.01 \times 10^5$
LA10	$1.68 \times 10^7 \pm 0.15 \times 10^7$	$0.98 \times 10^5 \pm 0.91 \times 10^5$
LA20	$1.51 \times 10^7 \pm 0.89 \times 10^7$	$0.84 \times 10^5 \pm 0.20 \times 10^5$

d e

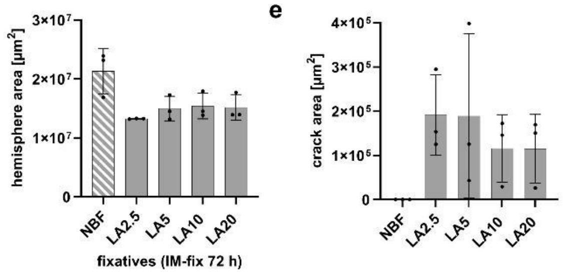

f

fixative (IM-fix 72 h)	hemisphere area (µm²) ± SD	crack area (µm²) ± SD
NBF	$2.13 \times 10^7 \pm 3.86 \times 10^7$	$0.00 \times 10^5 \pm 0.00 \times 10^5$
LA2.5	$1.33 \times 10^7 \pm 0.01 \times 10^7$	$1.91 \times 10^5 \pm 0.91 \times 10^5$
LA5	$1.50 \times 10^7 \pm 0.21 \times 10^7$	$1.89 \times 10^5 \pm 1.86 \times 10^5$
LA10	$1.54 \times 10^7 \pm 0.22 \times 10^7$	$1.16 \times 10^5 \pm 0.76 \times 10^5$
LA20	$1.52 \times 10^7 \pm 0.22 \times 10^7$	$1.15 \times 10^5 \pm 0.78 \times 10^5$

g h

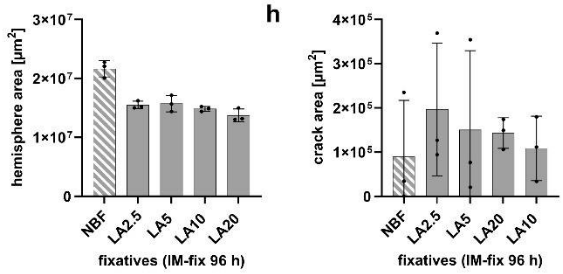

i

fixative (IM-fix 96 h)	hemisphere area (µm²) ± SD	crack area (µm²) ± SD
NBF	$2.16 \times 10^7 \pm 0.14 \times 10^7$	$0.90 \times 10^5 \pm 1.27 \times 10^5$
LA2.5	$1.55 \times 10^7 \pm 0.06 \times 10^7$	$1.97 \times 10^5 \pm 1.50 \times 10^5$
LA5	$1.57 \times 10^7 \pm 0.14 \times 10^7$	$1.51 \times 10^5 \pm 1.79 \times 10^5$
LA10	$1.48 \times 10^7 \pm 0.04 \times 10^7$	$1.08 \times 10^5 \pm 0.73 \times 10^5$
LA20	$1.37 \times 10^7 \pm 0.11 \times 10^7$	$1.43 \times 10^5 \pm 0.35 \times 10^5$

Supplemental 1. *Quantification of absolute crack areas after immersion fixation with the formalin-based control fixative (NBF) and lactic acid-containing test fixatives at different concentrations (LA2.5, LA5, LA10, and LA20) and different immersion durations: 24 hours (a-c), 72 hours (d-f), and 96 hours (g-i). The bar graphs in the first column (a, d, g) show mean hemisphere areas ± standard deviation (SD), while bar graphs in the second column (b, e, h) show mean crack areas ± SD for the corresponding hemispheres. Tables (c, f, i) summarize mean hemisphere areas and mean crack areas.*
