# Supplementary material for: Evaluation of lactic acid as a novel fixative for histological and neuroanatomical applications
Source: Sci Rep. 2026 May 11;16:15746. doi: 10.1038/s41598-026-51513-y (PMC13190837; doi:10.1038/s41598-026-51513-y)
Supplement: Supplementary file 2 — Supplementary Material 2 [file 41598_2026_51513_MOESM2_ESM.pdf]

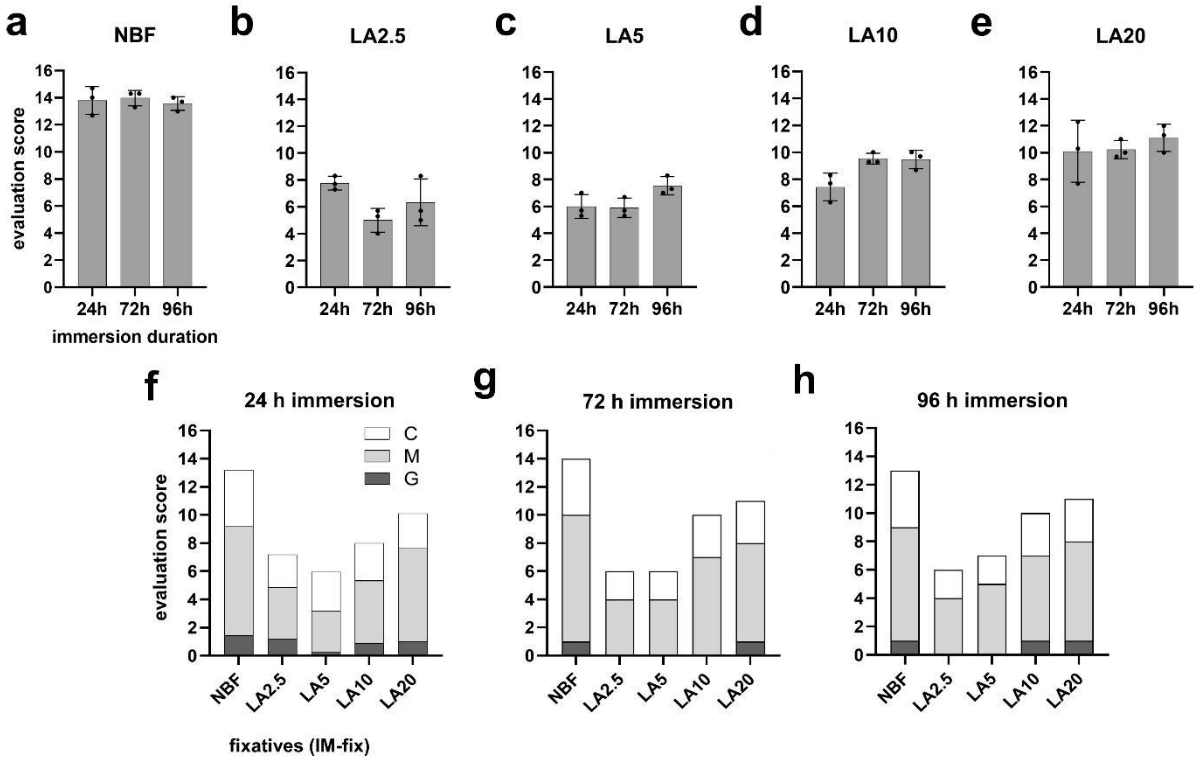

Supplemental 2. *Semi-quantitative evaluation of the histomorphological preservation after immersion with the formalin-based control fixative (NBF) and lactic acid-containing test fixatives at different concentrations (LA2.5, LA5, LA10, and LA20). Tissues were immersion-fixed for 24, 72, or 96 hours in separate, independent experiments. (a-e) Bar graphs show mean histomorphological evaluation scores ± standard deviation (SD) for each fixative, as assessed by n = 3 observers using one section per specimen and fixative (n= 3). (f-h) Stacked bar graphs show evaluation scores for each fixative and immersion duration, assigned to the subcategories listed in Table 1: general impression (G), histomorphology (M), and cytology (C), as assessed by n = 3 observers using one section per specimen and fixative (n= 3).*
