# Supplementary material for: Evaluation of lactic acid as a novel fixative for histological and neuroanatomical applications
Source: Sci Rep. 2026 May 11;16:15746. doi: 10.1038/s41598-026-51513-y (PMC13190837; doi:10.1038/s41598-026-51513-y)
Supplement: Supplementary file 3 — Supplementary Material 3 [file 41598_2026_51513_MOESM3_ESM.pdf]

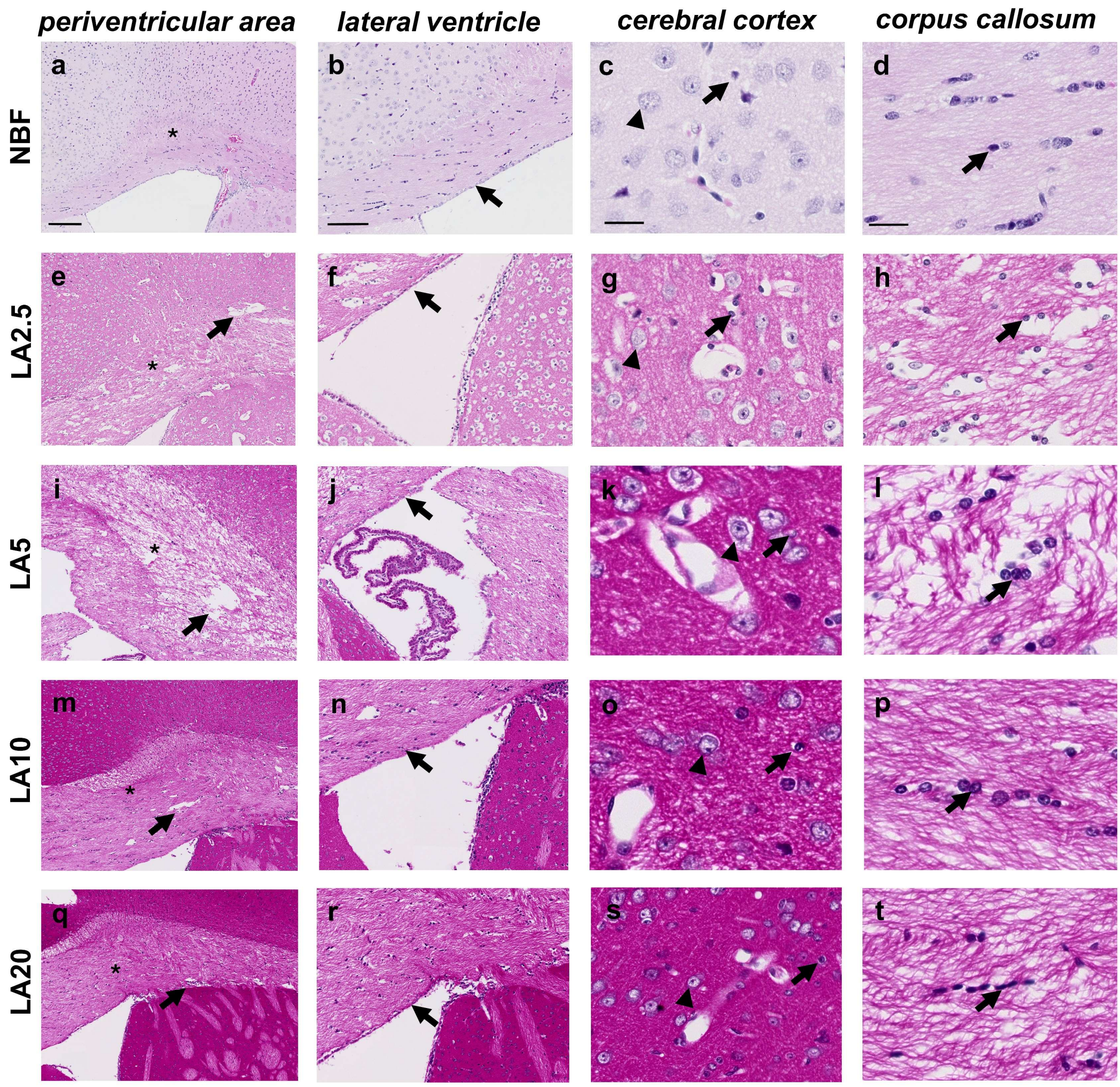

Supplemental 3. *Histomorphological preservation of murine brains after 72 hours of immersion fixation with the formalin-based control fixative (NBF) and lactic acid-containing test fixatives at different concentrations (LA2.5, LA5, LA10, and LA20), illustrated by H&E-stained frontal sections (Experiment 1; see Figure 6 for experimental design). The four columns display: (1) the periventricular area, (2) the lateral ventricle with adjacent corpus callosum and cortical/subcortical grey matter, (3) a high-power view of the cerebral cortex, and (4) a high-power view of the corpus callosum. Across all fixation conditions, the following features are indicated: loosening of white-matter fibres in the corpus callosum (stars in a, e, i, m, q); loosening of ventricular ependymal cells (black arrows in b, f, j, n, r); vascular structures (g, k, o, s); neuronal cell bodies in the cerebral cortex (black arrowheads in c, g, k, o, s), cells with glial-like morphology in the cerebral cortex (black arrows in c, g, k, o, s); and cells with glial-like morphology in the corpus callosum (black arrows in d, h, l, p, t). Scale bars: periventricular area, 200 µm; lateral ventricle, 100 µm; corpus callosum and cerebral cortex, 20 µm.*
