# Supplementary material for: Evaluation of lactic acid as a novel fixative for histological and neuroanatomical applications
Source: Sci Rep. 2026 May 11;16:15746. doi: 10.1038/s41598-026-51513-y (PMC13190837; doi:10.1038/s41598-026-51513-y)
Supplement: Supplementary file 5 — Supplementary Material 5 [file 41598_2026_51513_MOESM5_ESM.pdf]

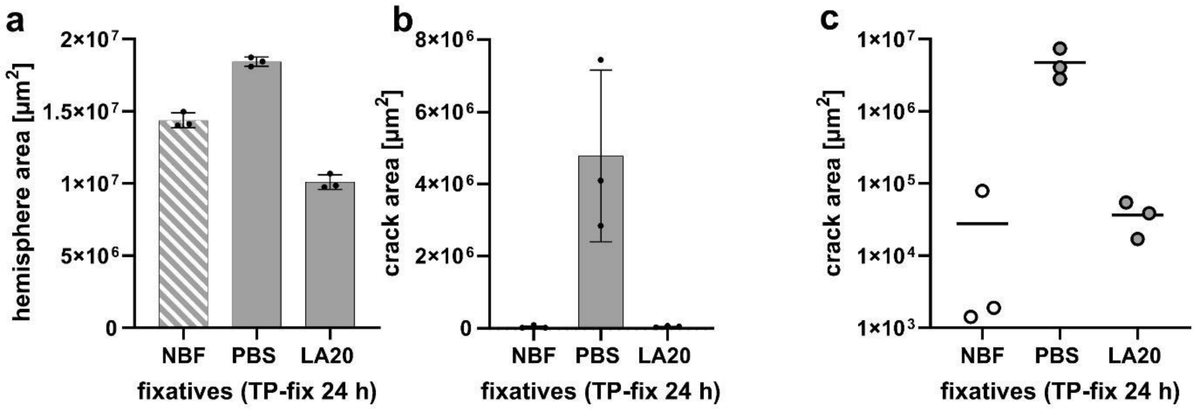

d

fixative (TP-fix 24 h)	hemisphere area (μm^2) ± SD	cracks area (μm^2) ± SD
**NBF**	$1.44\times10^7 \pm 0.05\times10^7$	$0.03\times10^6 \pm 0.04\times10^6$
**PBS**	$1.84\times10^7 \pm 0.03\times10^7$	$4.78\times10^6 \pm 2.38\times10^6$
**LA20**	$1.01\times10^7 \pm 0.05\times10^7$	$0.04\times10^6 \pm 0.02\times10^6$

Supplemental 5. *Quantification of crack areas relative to control fixatives (NBF and PBS) and test fixative containing 20% lactic acid (LA20) after transcardial perfusion followed by 24 hours of immersion post-fixation. (a) Mean hemisphere area ± standard deviation (SD) per fixative. (b-c) Mean crack area ± SD in the corresponding hemispheres. (d) Summary of mean hemisphere areas and mean crack areas ± SD.*
