# Supplementary material for: Evaluation of lactic acid as a novel fixative for histological and neuroanatomical applications
Source: Sci Rep. 2026 May 11;16:15746. doi: 10.1038/s41598-026-51513-y (PMC13190837; doi:10.1038/s41598-026-51513-y)
Supplement: Supplementary file 6 — Supplementary Material 6 [file 41598_2026_51513_MOESM6_ESM.pdf]

Table 2. Tested solution formulations for tissue fixation. [1]pH values slightly varied between single experiments. Experimental groups sorted by fixative solutions and its pH, fixation methods and immersion times (24 h, 72 h, and 96 h). IM-fix - immersion fixation, TP-fix - transcardial perfusion + immersion post fixation.

**Solution formulations**		**pH**	**IM-fix**	**TP-fix**
			**Immersion duration**	
NBF	Neutral buffered formalin	7.4	24h, 72h, 96h	24h
PBS	Phosphate-buffered saline	7.4	24h	24h
PBS_adj	Phosphate-buffered saline, pH adjusted	1.6	24h	-
LA2.5	Aqueous 2.5% LA solution	2.0-2.2[1]	24h, 72h, 96h	-
LA5	Aqueous 5% LA solution	1.9-2.0[1]	24h, 72h, 96h	-
LA10	Aqueous 10% LA solution	1.7-1.8[1]	24h, 72h, 96h	-
LA20	Aqueous 20% LA solution	1.4-1.6[1]	24h, 72h, 96h	24h
LA20_adj	Aqueous 20% LA solution pH adjusted	7.4	24h	-

Table 3. Dehydration steps and paraffin embedding.

**Step**	**Solution**	**Duration**
1	Running tap water	6 hours
2	50% ethanol (4°C)	overnight
3	70% ethanol	60 min
4	96% ethanol	60 min
5	96% ethanol	60 min
6	100% ethanol	60 min
7	100% ethanol	60 min
8	100% ethanol	60 min
9	100% xylene	60 min
9	100% xylene	60 min
10	100% xylene	60 min
11	Paraffin (56-58 °C)	60 min
12	Paraffin (56-58 °C)	60 min
13	Paraffin (56-58 °C)	1 – 6 h

Table 4. Deparaffinization steps.

**Steps**	**Solutions**	**Duration**
1	100 % Xylene	10 min
2	100 % Xylene	10 min
3	100 % Xylene	10 min
4	Xylene/Ethanol (70%/70%)	5 min
7	100% Ethanol	3 min
6	100% Ethanol	3 min
7	96% Ethanol	3 min
8	96% Ethanol	3 min
9	70% Ethanol	3 min
10	50% Ethanol	3 min
11	Distilled Water	3 min

Table 5. *Incubation times and differentiation steps in H&E staining.*

Steps	Solution	Duration
1	Mayer´s Hematoxylin	10 min
2	Water	5 min
3	HCl-ethanol 1%	4 sec
4	Water	15 min
5	Distilled Water	2 min
6	Eosin G	6 min
7	Water	30 sec

Table 6. *Dehydration steps of stained sections before coverslip-mounting.*

Steps	Solution	Duration
1	50% Ethanol	3 min
2	70% Ethanol	3 min
3	96% Ethanol	3 min
4	96% Ethanol	3 min
5	100% Ethanol	3 min
6	100% Ethanol	3 min
7	50% Ethanol / 50% Xylene	5 min
8	100% Xylene	10 min
9	100% Xylene	10 min
10	100% Xylene	10 min
11	Coverslipping	
