# Supplementary material for: Evaluation of lactic acid as a novel fixative for histological and neuroanatomical applications
Source: Sci Rep. 2026 May 11;16:15746. doi: 10.1038/s41598-026-51513-y (PMC13190837; doi:10.1038/s41598-026-51513-y)
Supplement: Supplementary file 7 — Supplementary Material 7 [file 41598_2026_51513_MOESM7_ESM.pdf]

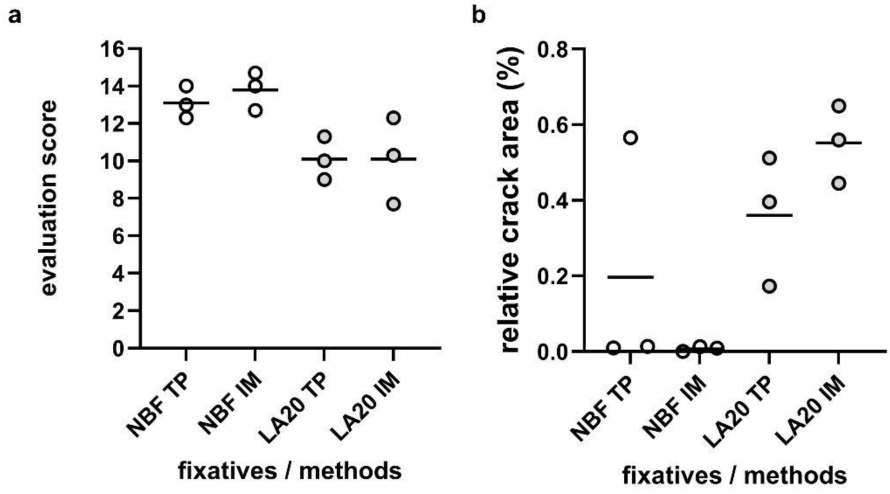

Supplemental 7. *Comparison of perfusion plus immersion (TP) and immersion-only (IM) fixation using neutral buffered formalin (NBF) and 20 % lactic acid (LA20) after 24 h of fixation. (a) Evaluation scores for individual specimens shown as dot plots with mean values indicated. (b) Relative crack area (%) of the corresponding hemispheres, shown as dot plots with mean values. Data are presented for descriptive purposes only, as TP and IM fixation were performed in separate experiments and are therefore not directly comparable.*
