# Supplementary material for: Evaluation of lactic acid as a novel fixative for histological and neuroanatomical applications
Source: Sci Rep. 2026 May 11;16:15746. doi: 10.1038/s41598-026-51513-y (PMC13190837; doi:10.1038/s41598-026-51513-y)
Supplement: Supplementary file 8 — Supplementary Material 8 [file 41598_2026_51513_MOESM8_ESM.pdf]

Table 7. *Semi-quantitative evaluation of the histomorphological preservation after immersion of murine brains with the formalin-based control fixative (NBF) and lactic acid-containing test fixatives at different concentrations (LA2.5, LA5, LA10, and LA20). Tissues were immersed for 24 hours. (a-e) Values represent the mean histomorphological evaluation scores ± standard deviation (SD) for each fixative, as assessed by n = 3 observers using n = 3 sections from different specimens per fixative.*

Immersion 24 h (IM-fix 24 h)

		NBF	LA2.5	LA5	LA10	LA20
1	**Overall integrity**	1.4 ± 0.4	1.2 ± 0.2	0.3 ± 0.0	0.9 ± 0.0	1.0 ± 0.7
2	**WM integrity**	2.0 ± 0.0	0.3 ± 0.0	0.1 ± 0.2	0.2 ± 0.5	0.9 ± 0.8
3	**WM precipitates**	1.4 ± 0.2	1.3 ± 0.0	1.3 ± 0.0	1.3 ± 0.2	1.7 ± 0.3
4	**Ependymal integrity**	1.9 ± 0.2	1.4 ± 0.2	0.9 ± 0.2	1.2 ± 0.0	1.1 ± 0.4
5	**Perineuronal spaces**	2.0 ± 0.0	0.6 ± 0.2	0.3 ± 0.0	0.7 ± 0.5	1.4 ± 0.4
6	**Perivascular spaces**	1.0 ± 0.3	0.3 ± 0.0	0.2 ± 0.2	0.4 ± 0.7	1.6 ± 0.5
7	**Nuclei differentiation**	2.0 ± 0.0	1.4 ± 0.4	1.4 ± 0.4	1.4 ± 0.4	1.2 ± 0.4
8	**Chromatin differentiation**	2.0 ± 0.0	1.0 ± 0.0	1.3 ± 0.3	1.2 ± 0.2	1.2 ± 0.4
	Score sum	13.8 ± 1.0	7.7 ± 0.3	6.0 ± 0.9	7.4 ± 1.0	10.1 ± 2.3

Table 8. *Semi-quantitative evaluation of the histomorphological preservation after immersion of murine brains with the formalin-based control fixative (NBF) and lactic acid-containing test fixatives at different concentrations (LA2.5, LA5, LA10, and LA20). Tissues were immersed for 72 hours. (a-e) Values represent the mean histomorphological evaluation scores ± standard deviation (SD) for each fixative, as assessed by n = 3 observers using n = 3 sections from different specimens per fixative.*

Immersion 72 h (IM-fix 72 h)

		NBF	LA2.5	LA5	LA10	LA20
1	**Overall integrity**	1.2 ± 0.2	0.0 ± 0.0	0.1 ± 0.2	0.4 ± 0.2	0.8 ± 0.2
2	**WM integrity**	1.8 ± 0.4	0.0 ± 0.0	0.3 ± 0.0	0.8 ± 0.0	0.6 ± 0.4
3	**WM precipitates**	2.0 ± 0.0	2.0 ± 0.0	2.0 ± 0.0	2.0 ± 0.0	2.0 ± 0.0
4	**Ependymal integrity**	1.9 ± 0.2	0.2 ± 0.4	0.3 ± 0.6	0.7 ± 0.2	0.4 ± 0.5
5	**Perineuronal spaces**	1.8 ± 0.2	0.6 ± 0.2	0.6 ± 0.4	1.4 ± 0.3	1.6 ± 0.2
6	**Perivascular spaces**	0.8 ± 0.3	0.2 ± 0.3	0.3 ± 0.6	1.7 ± 0.0	2.0 ± 0.0
7	**Nuclei differentiation**	2.0 ± 0.0	1.1 ± 0.2	0.8 ± 0.4	1.2 ± 0.4	1.4 ± 0.4
8	**Chromatin differentiation**	2.0 ± 0.0	0.8 ± 0.2	1.2 ± 0.4	1.4 ± 0.4	1.4 ± 0.4
	Score sum	13.9 ± 0.5	5.0 ± 0.4	5.9 ± 0.4	9.4 ± 0.5	10.2 ± 0.4

Table 9. *Semi-quantitative evaluation of the histomorphological preservation after immersion of murine brains with the formalin-based control fixative (NBF) and lactic acid-containing test fixatives at different concentrations (LA2.5, LA5, LA10, and LA20). Tissues were immersed for 96hours. (a-e) Values represent the mean histomorphological evaluation scores ± standard deviation (SD) for each fixative, as assessed by n = 3 observers using n = 3 sections from different specimens per fixative.*

Immersion 96 h (IM-fix 96 h)

		NBF	LA2.5	LA5	LA10	LA20
1	**Overall integrity**	1.2 ± 0.2	0.1 ± 0.2	0.6 ± 0.4	1.0 ± 0.0	1.0 ± 0.0
2	**WM integrity**	1.8 ± 0.4	0.3 ± 0.6	0.2 ± 0.4	0.8 ± 0.0	1.0 ± 0.0
3	**WM precipitates**	2.0 ± 0.0	2.0 ± 0.0	1.8 ± 0.2	1.8 ± 0.2	2.0 ± 0.0
4	**Ependymal integrity**	1.6 ± 0.5	0.8 ± 0.2	1.2 ± 0.4	0.7 ± 0.6	1.0 ± 0.0
5	**Perineuronal spaces**	2.0 ± 0.0	0.3 ± 0.6	1.0 ± 0.0	1.6 ± 0.5	1.9 ± 0.2
6	**Perivascular spaces**	1.0 ± 0.0	0.6 ± 0.4	0.8 ± 0.4	1.0 ± 0.0	1.1 ± 0.2
7	**Nuclei differentiation**	2.0 ± 0.0	1.0 ± 0.0	1.0 ± 0.0	1.3 ± 0.4	1.4 ± 0.4
8	**Chromatin differentiation**	2.0 ± 0.0	1.2 ± 0.4	1.0 ± 0.0	1.3 ± 0.4	1.4 ± 0.4
	Score sum	13.6 ± 0.5	10.9 ± 1.8	9.3 ± 0.7	6.3 ± 0.7	6.8 ± 0.8

Table 10. *Semi-quantitative evaluation of the histomorphological preservation after immersion of murine brains with the formalin-based control fixative (NBF) and test fixatives (PBS, PBS-Adjusted, LA20, and LA20-Adjusted). Tissues were immersed for 24 hours. Values represent the mean histomorphological evaluation scores ± standard deviation (SD) for each fixative, as assessed by n = 3 observers using n = 3 sections from different specimens per fixative.*

Immersion 24 h (pH adjustments)

		NBF	PBS	PBS_Adj	LA20	LA20_Adj
1	**Overall integrity**	1.0 ± 0.0	0.0 ± 0.0	1.1 ± 0.2	1.2 ± 0.8	0.0 ± 0.0
2	**WM integrity**	2.0 ± 0.0	1.0 ± 0.6	0.4 ± 0.5	1.6 ± 0.5	0.8 ± 0.2
3	**WM precipitates**	2.0 ± 0.0	2.0 ± 0.0	1.9 ± 0.2	2.0 ± 0.0	2.0 ± 0.0
4	**Ependymal integrity**	1.3 ± 0.3	0.9 ± 0.2	0.8 ± 0.2	1.1 ± 0.5	1.0 ± 0.0
5	**Perineuronal spaces**	1.4 ± 0.2	0.4 ± 0.2	0.4 ± 0.2	1.3 ± 0.7	0.3 ± 0.0
6	**Perivascular spaces**	0.8 ± 0.4	0.3 ± 0.0	0.6 ± 0.4	1.0 ± 0.4	0.3 ± 0.0
7	**Nuclei differentiation**	2.0 ± 0.0	0.1 ± 0.2	1.3 ± 0.0	1.4 ± 0.8	0.0 ± 0.0
8	**Chromatin differentiation**	2.0 ± 0.0	0.0 ± 0.0	1.1 ± 0.2	1.7 ± 0.6	0.3 ± 0.3
	Score sum	12.7 ± 0.3	4.6 ± 1.3	7.4 ± 0.8	11.4 ± 2.3	4.3 ± 0.3

Table 11. *Semi-quantitative evaluation of the histomorphological preservation transcardial perfusion, followed by immersion post-fixation for 24 hours with the formalin-based control fixative (NBF) or test fixatives (PBS, LA20) . Tissues were immersed for 24 hours. Values represent the mean histomorphological evaluation scores ± standard deviation (SD) for each fixative, as assessed by n = 3 observers using n = 3 sections from different specimens per fixative.*

Transcardial perfusion followed by 24 h immersion (TP-fix 24 h)

		NBF	PBS	LA20
1	**Overall integrity**	1.6 ± 0.4	0.0 ± 0.0	0.8 ± 0.4
2	**WM integrity**	1.6 ± 0.2	0.3 ± 0.0	1.2 ± 0.4
3	**WM precipitates**	1.6 ± 0.0	1.3 ± 0.0	0.3 ± 0.3
4	**Ependymal integrity**	1.7 ± 0.3	0.3 ± 0.0	1.1 ± 0.2
5	**Perineuronal spaces**	1.6 ± 0.2	0.0 ± 0.0	1.7 ± 0.0
6	**Perivascular spaces**	1.1 ± 0.2	0.2 ± 0.2	1.2 ± 0.2
7	**Nuclei differentiation**	2.0 ± 0.0	0.4 ± 0.2	1.9 ± 0.2
8	**Chromatin differentiation**	2.0 ± 0.0	0.0 ± 0.0	1.9 ± 0.2
	Score sum	13.1 ± 1.0	2.7 ± 0.0	10.1 ± 1.1
